# Development of a STandard reporting guideline for Evidence briefs for Policy (STEP): context and study protocol

**DOI:** 10.1186/s12961-022-00884-5

**Published:** 2022-07-23

**Authors:** Xuan Yu, Qi Wang, Kaelan Moat, Cristián Mansilla, Claudia Marcela Vélez, Daniel F. Patiño-Lugo, Yosef G. Abraha, Fadi El-Jardali, Racha Fadlallah, Jinglin He, Mohammad Kibria, Laura Boeira, Myeong Soo Lee, John N. Lavis, Yaolong Chen

**Affiliations:** 1 Evidence-Based Medicine Center, School of Basic Medical Sciences, Lanzhou University, Lanzhou, China; 2 Health Policy PhD Program, McMaster University, Hamilton, ON Canada; 3 McMaster Health Forum, McMaster University, Hamilton, ON Canada; 4 Department of Health Research Methods, Evidence and Impact, Faculty of Health Sciences, McMaster University, Hamilton, ON Canada; 5 University of Antioquia, Medellín, Colombia; 6 Faculty of Medicine, University of Antioquia, Medellín, Colombia; 7 Ethiopian Public Health Institute, Addis Ababa, Ethiopia; 8 American University of Beirut, Beirut, Lebanon; 9 Lanzhou University Institute of Health Data Science, Lanzhou, China; 10 Public Health Advisor, Health Intelligence and Research Department, Canadian Red Cross, Ottawa, Canada; 11 Instituto Veredas, Porto Alegre, Brazil; 12 Korea Institute of Oriental Medicine, Daejeon, Korea; 13 Africa Centre for Evidence, University of Johannesburg, Johannesburg, South Africa; 14 Research Unit of Evidence-Based Evaluation and Guidelines, Chinese Academy of Medical Sciences (2021RU017), School of Basic Medical Sciences, Lanzhou University, Lanzhou, China; 15 WHO Collaborating Centre for Guideline Implementation and Knowledge Translation, Lanzhou, China

**Keywords:** Evidence briefs for policy, Reporting guideline, Health and non-health sectors, STEP statement, Study protocol

## Abstract

**Background:**

Evidence briefs for policy (EBP) draw on best-available data and research evidence (e.g., systematic reviews) to help clarify policy problems, frame options for addressing them, and identify implementation considerations for policymakers in a given context. An increasing number of governments, non-governmental organizations and research groups have been developing EBP on a wide variety of topics. However, the reporting characteristics of EBP vary across organizations due to a lack of internationally accepted standard reporting guidelines. This project aims to develop a STandard reporting guideline of Evidence briefs for Policy (STEP), which will encompass a reporting checklist and a STEP statement and a user manual.

**Methods:**

We will refer to and adapt the methods recommended by the EQUATOR (Enhancing the QUAlity and Transparency Of health Research) network. The key actions include: (1) developing a protocol; (2) establishing an international multidisciplinary STEP working group (consisting of a Coordination Team and a Delphi Panel); (3) generating an initial draft of the potential items for the STEP reporting checklist through a comprehensive review of EBP-related literature and documents; (4) conducting a modified Delphi process to select and refine the reporting checklist; (5) using the STEP to evaluate published policy briefs in different countries; (6) finalizing the checklist; (7) developing the STEP statement and the user manual (8) translating the STEP into different languages; and (9) testing the reliability through real world use.

**Discussion:**

Our protocol describes the development process for STEP. It will directly address what and how information should be reported in EBP and contribute to improving their quality. The decision-makers, researchers, journal editors, evaluators, and other stakeholders who support evidence-informed policymaking through the use of mechanisms like EBP will benefit from the STEP.

*Registration* We registered the protocol on the EQUATOR network. (https://www.equator-network.org/library/reporting-guidelines-under-development/#84)

## Background

The need for evidence-informed policies and decisions to achieve the Sustainable Development Goals (SDGs) and other global-development targets is increasingly and widely recognized among policymakers, managers, providers, the public, researchers, and other stakeholders [1–4]. As an approach to ensuring policies and decisions are informed by the best available data and research evidence (e.g., systematic reviews), the concept of “evidence-informed policymaking” (EIP) includes helping clarify policy issues, frame options for addressing them, and identify implementation considerations [2, 5]. Evidence briefs for policy (EBP, sometimes referred to as “policy briefs” or “evidence briefs”) are increasingly used to support EIP in both health and non-health sectors and they typically have the following characteristics: (1) starting with a priority policy issue based on policymakers’ perspectives; (2) clearly presenting the nature and magnitude of the policy problem; (3) formulating possible policy options based on evidence about benefits, harms, costs, stakeholder views and experiences, and other elements; (4) combing global research evidence (prioritizing the best-available evidence syntheses related to the issue) and local evidence in a comprehensive and systematic way; (5) providing potential facilitators of and barriers to the implementation of selected options, and strategies to leverage facilitators and address barriers [6, 7]. Moreover, these documents are developed as inputs into deliberative dialogues in which participants use the pre-circulated evidence brief as a ‘jumping off’ point for deliberations, while bringing their views and experiences to bear on the issue in support of EIP.

Nowadays, more and more health and non-health EBPs are being prepared to address different issues by international organizations or academic groups, such as the Evidence-Informed Policy Network (EVIPNet). Moreover, related theories, methodologies, and practical handbooks about the development and usage of EBP are continuously evolving as experience grows.

However, the reporting format, characteristics, and lengths of EBP varies greatly across the organizations preparing them [8, 9]. Adam et al. analyzed 109 documents developed for policymakers and found little consistency in both the names used to label and describe “policy briefs” and in their contents. There were many variations in the feature and characteristics, including political/health system contexts, problems, options, implementation considerations, cost implications, methods, the quality of the evidence [8]. Most would not meet the above definition of an EBP. Zhang et al. analyzed 129 health-related EBPs published from 2016 to 2020 and also found there are differences in reporting details among different organizations, and observed many opportunities to improve their transparency and reliability [10]. Although some guidance on writing and reporting EBP are available [6, 8, 9], a comprehensive and widely accepted reporting guideline for EBP is needed to resolve some of the challenges described above.

Reporting guidelines are an important method to promote scientific research and developing guidelines for research-derived products such as EBP would similarly promote more systematic and transparent approaches to the development and reporting in these increasingly common documents. A clear, accurate, and complete EBP can not only help decision-makers and other stakeholders correctly understand the contents of the brief, but also help users make more evidence-informed decisions. Moreover, as one popular evidence-packaging document that addresses policy issues and provides policy options for policymakers, it is necessary to develop a widely accepted reporting guideline for EBP. The STandard reporting of Evidence briefs for Policy (STEP) project aims to develop a reporting guideline which will encompass a reporting checklist, a STEP statement & a user manual.

## Methods

We will refer to and adapt the methods recommended by the EQUATOR network [11]. Table 1 shows the detailed process with the proposed timeline.

**Table 1:** Adapted recommended-stages and project timeline

Stages	Actions	Tasks	Responsible groups	Timeline						
				2021			2022			
				Oct	Nov	Dec	Jan-Mar	Apr-Jun	Jul-Sep	Oct-Dec
Stage 1: Preparation	1	Identify the need for developing the STEP checklist based on a review of literature and documents	CT	✔						
	2	Obtain funding for the STEP project	CT	✔						
	3	Draft and register the protocol	CT; DP		✔					
	4	Identify the participants and establish the STEP working group	CT		✔	✔	✔			
Stage 2: Formulation	5	Generate the draft of STEP checklist with a list of potential items	CT				✔	✔		
	6	Conduct the modified Delphi process	CT; DP					✔	✔	
	7	Use the STEP checklist to evaluate a sample of policy briefs published in different languages	CT						✔	
	8	Approve the final STEP checklist	CT						✔	
	9	Develop the STEP statement and user manual	CT; DP						✔	✔
Stage 3: Implementation	10	Translate the STEP into other languages	CT; Relevant stakeholders							✔
	11	Test reliability through real world use and update the guideline	CT; DP; Relevant stakeholders	Every 3–5 years						

### Stage 1 preparation

#### Action 1. Identify the need for developing the STEP checklist

To identify the need for developing the STEP checklist, we searched the peer-reviewed literature and related documents on EBP and found there is no internationally accepted standard reporting checklist for EBP in health and non-health sectors. Moreover, our several interactions with different groups of decision-makers reaffirmed the need to develop a STEP checklist through a systematic and transparent process.

#### Action 2. Obtain funding

Our project is funded by the major project of the National Social Science Foundation of China: “Research on the Theoretical System, International Experience and Chinese Path of Evidence-based Social Science” (Project No. 19ZDA142). The funder will not interfere in the design of the study, in any aspect of data collection, analysis or interpretation, or in the writing and publication of the output.

#### Action 3. Draft and register the protocol

To enhance the transparency and quality of the STEP development process, we drafted a research protocol and registered it on the EQUATOR network (https://www.equator-network.org/library/reporting-guidelines-under-development/#84). The protocol will be published in the peer-reviewed journal and the development of STEP will be initiated after publication of the protocol.

#### Action 4. Identify the participants and establish the working group

The STEP working group consists of two subgroups: the Coordination Team (CT) and the Delphi panel (DP). We will collect both direct and indirect disclosures for all participants at the start of the STEP project and before publication.

1. Coordination Team (CT)

The members of the CT represent skills and experience covering project coordination, Delphi technique, development of EBP, reporting guidelines, guideline development, and implementation science. The roles of the CT are to coordinate the project; recruit the DP; draft the protocol and potential items; organize the Delphi process; collect and analyze data from the Delphi process; and draft the final checklist, statement, and explanatory document.

2. Delphi Panel (DP)

Considering the diversity of methodological expertise, gender equality and wide geographic representation, we will invite 10–20 individuals to the DP group, based on a review of the main authors in the field, as well as the recommendations from our working group. We will include experts with technical expertise in EBP development and implementation; EIP; health and non-health sectors; policy research; practice guideline development and implementation; knowledge translation; and reporting guidelines. To reflect the views and perspective of EBP target users and population, representatives of policymakers and other decision-makers (e.g., target users), researchers and the public (e.g., target population) will be also invited into this group. The roles of the DP group are to contribute to the protocol and development of checklist; and provide the comments on the final STEP checklist.

### Stage 2 formulation

#### Action 5. Generate a list of items for consideration in the Delphi process

We will conduct a literature search to extract potential reporting items. The search strategy, including the search sources and terms, will be developed with the help of an information scientist. We will mainly search six electronic databases: Web of Science, PubMed, EBSCO, PIE, Health Systems Evidence, and Social Systems Evidence. Also, we will additionally search related websites: 3IE International Initiative for Impact Evaluation, European Observatory on Health Systems and Policies, Evidence for Action (E4A), Evidence-Informed Policy Network (EVIPNet), Health Affairs, Health Systems Global (HSG), World Health Organization (WHO), and United Nations (UN). Moreover, we will search Google and Google Scholar for first 200 records and check the references of included studies and papers recommended by experts. The search strategy for PubMed is presented in Table 2.

**Table 2:** Search strategy for PubMed

Database	Search strategy
PubMed	#1 “policy brief*” [Title/Abstract] #2 “evidence brief*” [Title/Abstract] #3 “evidence brief for policy” [Title/Abstract] #4 “issue brief*” [Title/Abstract] #5 “citizen brief*” [Title/Abstract] #6 “research brief*” [Title/Abstract] #7 “evidence-informed policy brief” [Title/Abstract] #8#1–#7 OR

We will include the publications with the following characteristics: (1) publicly available, which clearly mentions one of EBP characteristics in both health and social science sectors between 2017 and 2021; and (2) publicly available research literature and/or documents related to EBP methodology, including development, reporting, use or implementation of EBP. We will exclude conference abstracts, editorials, commentaries, letters, opinions, correspondence, and news. Two reviewers will independently conduct the screening for literature and documents and the discrepancies will be resolved through discussions and consulting a third reviewer (if necessary).

We will develop a standardized form to extract the characteristics of each included document, including title, publication year, journal or development organization, and country focus (if applicable). We will extract and code the reporting characteristics (such as how to report problems, options, implementation considerations, methodology) from included documents, and conduct a thematic analysis based on the coding. Two reviewers will independently conduct the data extraction and coding for documents and the discrepancies will be solved through discussions and consulting a third reviewer (if necessary). During the process, iterative discussion and feedback among the research team members will be undertaken. Through the virtual meeting among research team, a list of potential items for reporting EBP will be developed and refined.

#### Action 6. Conduct the modified Delphi process

To achieve consensus on reporting items in the final STEP checklist, considering the possible attrition and participant fatigue [12], we will undertake three rounds of Delphi surveys, although there is a possibility that we may still be unable to reach consensus on some items during the third round. To reduce the response bias, we will accept a round as valid if there is a 70% response rate. Also, we will use several methods to enhance the response rate, including: setting up the expected time (usually within 10 days) for participants to return the questionnaire; sending a reminder letter to non-responders after 1 week; re-sending the questionnaire after 2 weeks. Our modified Delphi process is described in Table 3 [13–15].

**Table 3:** Organizing the Delphi process

Delphi Round	Key Points
Round 1	1. Circulate the introductory letter and background material 2. Send the Microsoft Word format questionnaire (Round 1 questionnaire) 3. Monitor attrition rate 4. Analyze the response and comments 5. Prepare Round 1 summary report and Round 2 questionnaire
Round 2	1. Circulate Round 2 questionnaire with consensus results from Delphi Round 1 2. Monitor attrition rate 3. Analyze the response and comments 4. Prepare Round 2 summary report 5. If the consensus is reached, we will terminate Delphi and prepare the final report; if not, we will proceed to Round 3
Round 3	1. Prepare and circulate Round 3 questionnaire with consensus results from Delphi Round 2 2. Monitor attrition rate 3. Analyze the response and comments 4. Prepare and circulate Round 3 summary (e.g., final) report 5. Other round will not be continued although some items cannot be reached consensus

The questionnaire will be piloted among the CT. The content and format of the questionnaire will be adjusted through discussion. We will send the initially prepared items in Microsoft Word format to score each item of the initial STEP checklist and collect suggestions and modifications. We will use a 7-point scale (1 = strongly disagree, 2 = disagree, 3 = disagree slightly, 4 = neither disagree or agree, 5 = agree slightly, 6 = agree and 7 = strongly agree) to rate each potential item [14–16].

Participants will be provided with the opportunity to suggest refinements and modifications to each of the candidate items and to suggest additional items not addressed in the list. The responses for each round will be analyzed using Microsoft Excel. For the scored quantitative data, we calculate the median score. The definition of agreement and consensus are presented in Table 4. Based on the response for each round of Delphi: (1) items with ‘agreement’ will be included in the final version and be removed from subsequent rounds; (2) items with ‘disagreement’ will be deleted; (3) items which are rated as ‘ambivalent’ or where there is no consensus will be modified according to the comments from the expert panel and the discussion of the research team, then will be included in the next Delphi round. Additional items nominated by the panel will be reviewed by the research team and reworked to align with the style and format of the other candidate items [13, 15]. According to the Delphi results, we will draft a checklist of included reporting items for EBP.

**Table 4:** Definition of consensus

Definition	Criteria
Definition of agreement with an item	When median score of the item ≥ 6
Definition of disagreement with an item	When median score of the item ≤ 3
Definition of ambivalence towards an item	When median score of the item is from 3 (exclusive) through 6 (exclusive)
Definition of no consensus within the group	All other types of responses

#### Action 7. Use the STEP to evaluate published policy briefs

After the second or third round of Delphi surveys is completed, we will generate a draft version of the STEP checklist. To validate the utility, applicability, and reliability of the STEP checklist, we plan to use the STEP checklist to evaluate a sample of policy briefs published in different languages (such as Chinese, Spanish, Korean, Portuguese, Arabic) in the last three years. Through the evaluation of a sample of EBP, we hope to identify current deficiencies and weaknesses in the tool, record them and use them to inform the final updates and improvements to the STEP checklist. CT will collect and analyze results, and discuss them with DP in the next phase (e.g., Action 8).

#### Action 8. Approve the final checklist

Due to the COVID-19 pandemic, we will organize a virtual (rather than in-person) conference including representatives of the CT and DP groups to present and discuss the results of the Delphi process and evaluation results, then will draft the final checklist. We will send it to the representatives of the CT and DP for their review to ensure accuracy.

#### Action 9. Develop the STEP statement and user manual

The CT will develop STEP statement and user manual, including rationale for developing the checklist, development process, and the checklist. A purposive sample of participants will be recruited to for pilot testing. We will not limit participants according to their experience and exposure to EBP development and/or use, which will help us better estimate of the usability of the guideline among its expected users. We will seek feedback by pilot testing, which will include contacting individuals and groups involved in developing EBP, asking them to use the checklist to inform the preparation of their EBP, followed by the collection of feedback (by online survey) from them about any further revisions that need to be made to the guideline. We will publish the STEP in a peer-reviewed journal.

### Stage 3 implementation

#### Action 10. Translate the STEP into other languages

We will welcome and collaborate with other EBP developers who want to translate this guideline into other languages.

#### Action 11. Test the reliability through real world use and update the guideline

We will test the reliability of the checklist through continuous collection, comparison and analysis of user data using a feedback mechanism by emails. Moreover, we plan to update the STEP every 3–5 years.

## Discussion and conclusion

The main output of our project is a STandard reporting guideline of Evidence briefs for Policy (STEP), which will encompass a checklist of reporting items for EBP in health and non-health sectors, a STEP statement, and a user manual. The STEP will directly address what and how information should be reported in EBP. We also hope to reach a consensus on the term of EBP in this project. Moreover, policy makers, decision-makers, researchers, users, journal editors, and evaluators will benefit from the STEP statement.

The main strength of our research is the systematic, transparent and rigorous methods that will be applied to identify EBPs not only in the health sector, but also in the social sciences. The multi-layered approach to developing a checklist through systematic literature review followed by refining the list through a Delphi process will enhance the transparency and credibility of our findings. STEP working group members have years of experience in developing and evaluating EBP and publishing them (or studies evaluating them) in academic journals. However, there some challenges may exist: first, EBP that are not in the public domain will not be included for obvious reasons; second, we do not plan to conduct a quality assessment for all included EBP because there are no appraisal tools widely accepted.

We will collaborate with some organizations, institutions, and groups who are preparing and/or using EBP to implement STEP, and continuously seek feedback from developers, users, and stakeholders. We will disseminate the STEP by publishing into a peer-reviewed journal, presenting to relevant stakeholders and translating into different languages. We will update the checklist based on the continuous feedback and new relevant research evidence.

## Data Availability

Not applicable.

## Abbreviations

3IE: International Initiative for Impact Evaluation
CT: Coordination team
DP: Delphi panel
E4A: Evidence for action
EBP: Evidence briefs for policy
EIP: Evidence-informed policymaking
EQUATOR: Enhancing the QUAlity and Transparency Of health Research
EVIPNet: Evidence-Informed Policy Network
HSG: Health systems global
PEERSS: Partnership for evidence and equity in responsive social system
SDGs: Sustainable development goals
STEP: STandard reporting guideline of Evidence briefs for Policy
UN: United Nations
WHO: World Health Organization
